# Maternal hair selenium levels as a possible long-term nutritional indicator of recurrent pregnancy loss

**DOI:** 10.1186/1472-6874-13-40

**Published:** 2013-10-22

**Authors:** Viju V Thomas, Robert Knight, Stephen J Haswell, Stephen W Lindow, Zephne M van der Spuy

**Affiliations:** 1 Department of Obstetrics and Gynaecology, Faculty of Health Sciences, Groote Schuur Hospital, University of Cape Town, 7937 Cape Town, South Africa; 2 Department of Chemistry, University of Hull, Hull, UK; 3 Department of Obstetrics and Gynaecology, University of Hull, Hull Royal Infirmary, Hull, UK

**Keywords:** Selenium, Recurrent pregnancy loss, Miscarriage, Micronutrients, Hair

## Abstract

**Background:**

Approximately 1% of all couples trying to conceive will suffer from recurrent pregnancy loss (RPL). Nutritional deficiencies have been postulated as a possible cause of RPL and in particular, selenium deficiency has been associated with reproductive failure in animal studies and more recently, in some human studies. This study was undertaken to assess the maternal hair selenium levels in women with RPL without an identified cause and to compare these results with those of women with successful reproductive histories.

**Methods:**

Twenty four patients with RPL and twenty four control subjects with at least one successful pregnancy and no pregnancy failures, who were matched for age and ethnicity, were recruited. A questionnaire was completed, which included demographic and social information and a dietary history. Hair samples were collected and analyzed for selenium content by inductively coupled plasma mass spectrometry.

**Results:**

The control subjects had a higher mean income and had completed more years of education compared with the RPL patients. There was no significant difference in the intake of selenium rich foods between the 2 groups. The patients, however, consumed significantly more fruit, cheese, potatoes and chocolate than the controls. The median (range) selenium content was 0.80 ppm (0.19-4.15) and 0.68 ppm (0.43-3.76) in patients and controls respectively (Mann Whitney *U* test 209.5 p = 0.74).

**Conclusions:**

While there were significant differences in the 2 groups with regard to resources, education and diet our results show that hair selenium concentrations and dietary selenium intake, were similar in the two groups. Both groups had low levels of this important element.

## Background

Miscarriage is the most common complication in pregnancy with an incidence of 12-31% [1]. In South Africa it contributes to 30% of “pregnancy related sepsis” and is directly responsible for 3.5% of all maternal deaths [2]. Recurrent pregnancy loss (RPL) is defined as the loss of three or more consecutive pregnancies before 20 weeks gestation and is estimated to affect 1% of all couples trying to conceive [3,4].

Malnutrition is a global problem. In developed countries it is often related to inappropriate diets or eating disorders while in developing countries, chronic ill health and food scarcity contribute to malnutrition [5]. Micronutrient deficiencies associated with malnutrition, such as selenium, copper, zinc and iron have been implicated in miscarriage and selenium deficiency has been identified as a possible cause for RPL in several studies [6-10].

Selenium is a key component of a number of selanoproteins, of which the best known is the antioxidant glutathione peroxidase, which contributes to the delicate balance between pro-oxidants and anti-oxidants, a critical aspect for survival in aerobic organisms.

The concentration of selenium in the soil is reflected in the plants and the animals that feed on these plants. Reproductive failure as a consequence of selenium deficiency has been demonstrated in a number of animal and agricultural studies. Soil selenium levels differ between countries and local studies in herbivores have demonstrated that the selenium concentration in South African soils is low [11]. Humans obtain selenium through their diet. Studies from the UK have demonstrated that selenium concentrations are highest in foods like Brazil nuts (245 ug/100 g), kidney (146 ug/100 g), crab meat (84 ug/100 g) and liver (42 ug/100 g). Other sources of selenium include bread, cereals, meat, poultry and fish [12].

A number of women with RPL have no identifiable cause for their pregnancy failure. An expert review article suggests that reduced antioxidant protection may contribute to early pregnancy loss [13]. Given the data on selenium, the findings of other studies and the suggested selenium deficiency in the Western Cape, this study was undertaken to determine whether selenium deficiency was present in women with RPL and to compare this group to women with an uncomplicated obstetric/pregnancy history. In view of the proven value of determining micronutrients and other substances in hair, the hair selenium status was measured as a reflection of long-term nutritional status.

## Methods

Our aim was to assess the association between RPL and selenium deficiency in a population living in a low selenium environment.

This study was approved by the Research Ethics Committee of the Faculty of Health Sciences, University of Cape Town (Research Ethics Committee Reference no: 119/94 and 041/2006). Written informed consent was obtained from all participants. This case–control study included a total of 24 patients and 24 control subjects. RPL was defined as women with 3 consecutive first trimester miscarriages or 2 second trimester miscarriages. Patients consisted of non-pregnant women, not using hormonal contraception who suffered from RPL. All patients were recruited from the Reproductive Failure Clinic at Groote Schuur Hospital, Cape Town, and had no identifiable pathology when screened for known causes of RPL, e.g. anatomical defects, chromosomal abnormalities, endocrinopathies, immunologic and thrombotic pathology.

Control subjects were healthy, non-pregnant women with at least one live child who were not using hormonal contraception and had not been pregnant during the previous 6 months. These controls and subjects were matched for age (±2 years) and ethnicity. Controls were recruited from women attending our outpatient clinics and staff members who fulfilled the criteria. Inclusion and exclusion criteria are tabulated in Table 1.

**Table 1:** Inclusion and exclusion criteria

***Inclusion criteria***		***Exclusion criteria***	
**CASES**	**CONTROLS**	**CASES**	**CONTROLS**
Non pregnant women	Non pregnant women	Currently pregnant or lactating	Currently pregnant or lactating
Three consecutive 1st trimester miscarriage or two 2nd trimester losses	Uncomplicated obstetric history	Cause for RPL identified.	Any underlying medical condition that may cause RPL
No live births after miscarriages	One or more successful pregnancies	Use of hormonal contraception in preceding 6 months	Use of hormonal contraception in preceding 6 months
No diagnosis for RPL established	N/A	Not living in Cape Town for more than 2 years	Not living in Cape Town for more than 2 years
No hormonal contraception in preceding 6 months	Not on hormonal contraception		

A datasheet was completed for each recruit incorporating demographic details, proximity to industrial areas, types of dwelling/roofing, cooking utensils, contraceptive use, personal habits (smoking and alcohol consumption), dietary history, hair products/treatments and reproductive history. We sought information regarding diet, hair treatments and contraception for 6 months prior to sampling. Hair was sampled from the nape of the neck, just above the hair line and marked proximally to identify the newest growth. The RPL and control subjects were compared using a chi square or *t* test as appropriate.

### Analysis of hair samples

Sharp surgical scissors were used to cut hair from the nape of the neck. All samples were stored in a plastic container at room temperature and couriered to the University of Hull, Department of Chemistry, where preparation and analysis was performed and reported. The samples were washed with lipsol detergent in pure water to remove surface debris, then air dried overnight. The hair was weighed and put into Teflon digestion vessels with 0.5 ml nitric acid, sealed and left overnight. They were then placed in a microwave oven (CEM MARS Xpress) and heated at high temperature, then cooled to room temperature. Once opened the contents were diluted in 5 ml water and analysed using the Perkin Elmer Elan DRCII inductively coupled plasma mass spectrometry (ICPS) instrument. Selenium was calibrated at 0, 1, 5, 10, 20 parts per billion (ppb), which gave linear calibrations. The measured concentrations of selenium were then calculated to give the amount in the original dry hair samples by multiplying the measured concentration by the dilution factor. Any dilutions of the digests were allowed for at this point. Changing the units gave values in ppm, or micrograms per gram of original dry solid. Gaps in the tables were filled in by recalculation of the data in the Elan software sample by sample. The limit of detection was 1.016 ppb. Except for 4 values (3 RPL and 1 control), the measured concentrations of selenium were all above these values.

## Results

Twenty four women were recruited to each group (patients and controls). All the patients who were invited agreed to participate, while in the control group, out of 31 potential subjects, 7 were unsuitable and 24 satisfied all criteria and agreed to participate. Subjects gave written informed consent. Cases and controls were matched for ethnicity. Each group had 17 participants of mixed race origin, 6 black African and 1 Indian subject. Table 2 presents the general characteristics of the two groups. The mean ages were 32.5 years (RPL) and 33 years (control group).

**Table 2:** Background patient characteristics

**Characteristics**	**Recurrent miscarriage n = 24**	**Control n = 24**	**P**
Age(y)	32.5 (5.62)	33 (5.87)	0.775
BMI (kg/m^2^)	31.9 (21.8)	28.5 (6.3)	0.473
Education (y)	10.0(3.12)	14.9(4.23)	0.374
Gravidity	4.9 (1.66)	2.3 (1.22)	0.001
**Household income/Rand per month**	**R. 6147.55 (8022.6)**	**R. 14761 (11351.44)**	**0.006**
Miscarriages	3.7 (1.2)	0 (0)	0.001
Weight gain(kg)	1.9(3.43)	3.2(3.66)	0.214
Weight loss(kg)	1.0(1.79)	0.7(1.60)	0.87

Mean (SD).

The only significant differences between the two groups were obstetric outcomes, as would be expected, and household income. The mean number of miscarriages in the RPL group was 3.7 ±1.2. There was a significantly higher monthly household income among control subjects (mean income R6221 (SD 8195) v R14762 (SD 11352) p < 0.006) in RPL and controls respectively. Although not statistically significant, analysis of educational achievements showed that the controls had an average of 4.9 years more of formal education than the patients.

The results of the dietary questionnaire are presented in Table 3. Comparison of the consumption of selenium rich foods namely nuts, liver and kidney did not show any significant difference between the groups. In the RPL group, there was a statistically significant increase in weekly consumption of cheese [4.85 (sd.9.3) vs. 0.44 (sd.0.64) P = 0.02], fruit [4.88 (sd. 6.9) vs. 1.46 (sd.1.06) P = 0.025], chocolate [1.9 (sd.2.65) vs. 0.54 (sd.0.72) P = 0.016] and potato [4.58 (sd.4.9) vs. 0.98(sd.0.93) P = 0.001]. There was no difference in the consumption of alcohol or of smoking. All women were asked about the use of anti-dandruff shampoo and hair colouring agents. In the RPL group 62.5% used hair colouring agents, including one patient who had used henna products within the preceding 6 months, compared with 45.8% in the control group (P = 0.424). A review of shampoo brands used by our subjects revealed that none contained selenium, but the henna product did have selenium and this was reflected in the results of her hair analysis.

**Table 3:** Results of dietary questionnaire

**Food, drink and habits**	**Recurrent miscarriage n = 20**	**Control n = 23**	**P**
**Alcohol (units/day)**	**0.7(1.7)**	**0.4(1.2)**	**0.957**
**Bread (slices/day)**	**3.2(2.0)**	**3.0(2.0)**	**0.757**
**Cereals (bowls/day)**	**0.6(0.7)**	**0.6(0.5)**	**0.528**
**Ceylon tea (cups/day)**	**1(2.1)**	**1.0(1.3)**	**0.921**
***Cheese (helping/week)***	***4.9(9.3)***	***0.4(0.6)***	***0.026***
***Chocolate (bars/week)***	***1.9(2.7)***	***0.5(0.7)***	***0.016***
**Coffee (cups/day)**	**1.8(2.4)**	**2(1.5)**	**0.73**
**Cream (helpings/week)**	**0.1(0.3)**	**0.04(0.2)**	**0.575**
***Fruit (pieces/week)***	***4.9(6.9)***	***1.5(1.1)***	***0.025***
**Liver/kidney (helping/week)**	**0.6(1.4)**	**0.1(0.2)**	**0.129**
**Nuts (grams/day)**	**10.4(29.3)**	**7.9(17.4)**	**0.728**
**Peas/beans(helpings/day)**	**0.9(1.9)**	**0.7(0.5)**	**0.627**
***Potato (helping/week)***	***4.6(4.9)***	***1.0(0.9)***	***0.001***
**Rooibos tea (cups/day)**	**0.7(1.0)**	**0.4(0.9)**	**0.317**
**Smoking (cigarettes/day)**	**5.2(7.3)**	**2.6(4.8)**	**0.550**

### Selenium levels in hair

**Figure 1:**
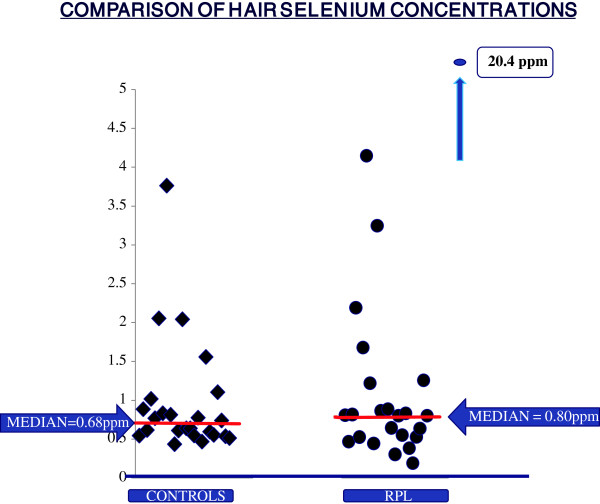
Selenium concentrations on vertical axis in ppm.

Table 4 represents our findings of selenium levels in hair. Hair selenium levels were not normally distributed and log selenium levels were also not normally distributed therefore a non-parametric analysis was undertaken. The median (range) selenium content was 0.80 ppm (0.19-4.15) and 0.68 ppm (0.43-3.76) in patients and controls respectively (Mann Whitney *U* test 209.5 p = 0.74). There was no significant difference in hair selenium levels between the two groups. Figure 1 demonstrates that there is an outlier with a very high selenium concentration of 20.4 ppm. A review of this patient’s questionnaire revealed that she was the only subject who had used henna hair products in the preceding six months and this result was excluded from the analysis. This patient fell into the lower income category and her dietary history did not show any difference in any food consumption. The concentrations from 3 RPL and 1 control subject fell below the limit of detection. These 4 values together with the one outlier were excluded from the statistical analysis.

**Table 4:** Hair selenium concentrations

	**Selenium (ppm) median**	**Range (ppm)**
RPL n = 20	0.80	0.19-4.15
CONTROLS n = 23	0.68	0.43-3.76

Mann Whitney *U* test 209.5 (p = 0.74). (Analysis excluding 5 women, 3 RPL and one control whose selenium concentrations fell below the limit of detection and one outlier in the RPL group who had used henna hair products).

Because the control group had significantly better incomes, the income and hair selenium level was correlated across the combined group of women. Hair selenium concentrations were not significantly correlated with income (Pearson correlation coefficient −0.183, p = 0.219).

## Discussion

The aetiology of RPL is probably multi-factorial and may include nutritional and micronutrient deficiencies.

Undernutrition and malnutrition are recognised as having a potential impact on reproductive performance. The women who attend our Reproductive Failure Clinic often do not have an identified cause for their pregnancy loss, which makes future management and counselling difficult. Nutritional deficiencies have been identified as causes of failure of both fertility and fecundity. Animal work makes a compelling case for a role for selenium in successful reproduction and recent studies in women with RPL have suggested that selenium deficiency may contribute to RPL [10,14-16]. Our study was undertaken in an attempt to identify the selenium status in women with RPL of unknown cause and compare this to those in women with successful reproductive histories. If selenium is deficient in our clinic population replacement therapy may prove helpful in affected patients.

Using hair as the tissue for study offers an opportunity to identify chronic/long term selenium status. Forensic science has utilized hair for analysis of chronic drug exposure, pesticide exposure and post mortem toxicology and these methods are validated and reproducible [17-21]. Al- Kunani et al., in a study undertaken in Hull, UK, using blood and hair, demonstrated selenium deficiency in the RPL group in hair samples but not blood samples [10].

As hair selenium levels may be affected by use of hair products, such as certain anti-dandruff shampoos and henna [19,21], we reviewed all products used by subjects in the preceding 6 months. One patient was using henna hair dye, and this is reflected in the abnormally high selenium levels in her hair. The analysis was done after excluding this outlier. Further confounding factors were taken into account such as environmental exposure from roofing, type of housing, proximity to industrial areas, access to running water and cooking utensils. All subjects had been living in Cape Town for at least two years and comparison between the two groups showed no difference in environmental exposure.

Studies have demonstrated a positive correlation between serum estradiol levels and glutathione peroxidase and we therefore excluded any women who had used hormonal contraception over the preceding 6 months [22,23].

The two groups differed significantly in family incomes, with the median income of the controls almost four times that of the patients (R12850 vs. R3450). There was a non-significant difference in their education levels, where the controls studied on average 4.9 years more than patients. This result reflects the different populations selected for each group, where control subjects included appropriate hospital staff members. Although not intended, the difference between the two groups may mean that controls had more access to dietary information, enabling them to eat healthier, and possibly consume food types with higher selenium content. Comparison of the selenium rich foods, however, showed no difference in their intake, possibly suggesting no advantage among the control subjects in terms of dietary information supplied to them, despite their educational and economic advantages.

Patients and controls had different dietary habits, but not with regard to selenium rich foods. Obviously in the controls, the levels may have been different during their successful pregnancies but there is no way of ascertaining this and it seems likely that their diet was fairly constant over time.

The results show a significantly higher intake in the RPL group of potato, cheese, fruit and chocolate and these foods are relatively low in selenium. In South Africa, fruit is readily affordable and potato is relatively cheap and more portions was eaten by patients per week. A fungus, Phytophthora infestans, was identified as the cause for potato blight in the Irish Famine of 1844 and although there is no evidence in the literature that there is a direct link to miscarriage however the possibility that this fungus may cause miscarriage should be considered.

The selenium levels from the study by Al Kunani et al. and this study were analysed in the same laboratory at the University of Hull, and in comparison with the UK (Al Kunani [10]) results, women in South Africa had comparatively higher selenium concentrations, possibly suggesting higher selenium in South African soils and foods.

In contrast to our findings, Ejezie et al. [24] recruited 40 women (n = 120) in each trimester and 35 control subjects and measured serum selenium concentration during pregnancy and postpartum and found significantly decreased selenium concentrations in pregnancy, when compared to non pregnant controls. The authors concluded that selenium supplementation might be indicated in pregnancy.

## Conclusion

Micronutrient deficiency has been documented in a number of reproductive disorders and in a population with absolute and relative nutritional compromise. This study did not demonstrate selenium differences in the two groups studied but a nutritional component, still to be defined, in RPL is still a possibility particularly when one reviews the socioeconomic deprivation in many of our communities. In our opinion selenium supplementation in RPL cannot be recommended until more research has been performed.

Future research possibilities include exploring and comparing different tissue types to evaluate selenium and other micronutrients and employing prospective studies to evaluate selenium concentrations in RPL.

## Competing interests

The authors declare that they have no competing interests.

## Authors’ contributions

VT collected the data and drafted the manuscript. RK and SH carried out the laboratory work. ZVDS and SL participated in the design of the study. SL performed the statistical analysis. SL and ZVDS conceived of the study and participated in its design and coordination and helped to draft the manuscript. All authors read and approved the final manuscript.

## Pre-publication history

The pre-publication history for this paper can be accessed here:

http://www.biomedcentral.com/1472-6874/13/40/prepub

## Condensation

This study measured hair selenium levels in women with recurrent miscarriage and compared them to those with uncomplicated reproductive histories in a tertiary unit in South Africa. While differences in dietary habit and socioeconomic status were demonstrated, selenium levels in the two groups were not significantly different.
